# A quality improvement program for adolescents with cystic fibrosis: focus on psychosocial skills

**DOI:** 10.1186/s13023-017-0747-5

**Published:** 2018-02-08

**Authors:** Michele Gérardin, Anne Pesle, Dominique Pougheon-Bertrand, Pilar Léger, Céline Vallet, Tiphaine Bihouee, Valérie David

**Affiliations:** 1 Pediatric CF Center, R, Debré University Hospital, AP-HP, Paris, France; 2 LEPS EA3412, Sorbonne Paris Cité University, Bobigny, France; 3 Pediatric CF Center, Nantes University Hospital, Nantes, France; 4 Quality Department, Nantes University Hospital, Nantes, France

**Keywords:** Cystic fibrosis, Quality improvement program, Therapeutic patient education, Transition for adolescents, FEV1

## Abstract

**Background:**

The two pediatric cystic fibrosis centers (CFCs) in Paris (Robert Debré) and Nantes, France, have been developing therapeutic patient education (TPE) programs since 2006 and have been engaged in the pilot phase of the quality improvement program (QIP) named the Hospital Program to Improve Outcomes and Expertise in Cystic Fibrosis (PHARE-M) since 2011. The objective was to improve the FEV1 of the cohort of adolescents to prepare them for their optimal transition to an adult CFC.

**Methods:**

The two CFCs formed a multidisciplinary quality team and used the analysis of causes of insufficient respiratory function taking into account the adolescents’ psychosocial factors. At the Nantes CFC, the approach was centered on adolescents’ body image and their motivation to take care of themselves by assigning specific aspects of patient follow-up to each professional in the team. At R. Debré, an individual cause-and-effect diagram identified for each patient the medical and psychosocial factors that could account for insufficient respiratory function. Personalized actions were offered to each patient.

**Results:**

In 2014, the median FEV1 (Forced Expiratory Volume in 1 Second) of the adolescent cohort exceeds 90% at the 2 CFCs (Nantes and R. Debré). Between 2011 and 2014 both centers improved their ranking for FEV1% in adolescents in the Registry histograms. At R. Debré, the personalized process allowed to reinforce equality of care, offering to all the opportunity to benefit from TPE sessions and coaching with an adapted physical activity teacher. The psychologist developed a specific tool to support the patient-centered process.

**Conclusion:**

The link between TPE and QIP was strong at our two centers enhancing patient centered care and targeting an optimal transition to an adult program.

## Background

The prognosis of cystic fibrosis is mainly associated with respiratory status. In the current lack of curative treatment, the objective must be to maintain good respiratory function over time. During adolescence, patients are more likely to see a decrease in their FEV1, the main indicator of their respiratory status [1].

As in all chronic diseases, adolescents with cystic fibrosis have more or less significant difficulties in complying with treatments and finding motivation to take care of themselves [2]. These difficulties may have repercussions on their respiratory status [3].

Adolescents with cystic fibrosis have generally been followed up for many years in cystic fibrosis centers (CFCs), even since birth since newborn screening has been generalized in France in 2002. They have progressively acquired a great deal of knowledge on the disease and the treatments, and are gradually gaining autonomy, both as regards their treatments and their life plans. They transition to an adult program between ages 18–20.

In 2007, the French National Authority for Health (HAS) published recommendations for therapeutic patient education (TPE) (http://www.has-sante.fr/portail/upload/docs/application/pdf/etp_-_guide_version_finale_2_pdf.pdf). The TPE definition from the World Health Organization in 1998 [4] is: “helping patients acquire or maintain the competencies they need to manage as well as possible their lives with a chronic disease.” TPE programs require an authorization issued by the French Regional Health Agencies (ARS), renewed according to a quadrennial evaluation based on the guidelines prepared by the HAS [5].

The two pediatric CFCs in Paris (Robert Debré) and Nantes have developed similar therapeutic education programs allowing children and their parents to acquire and evaluate skills during individual and group sessions.

In 2009, the French Cystic Fibrosis Registry’s report by center indicated that the median FEV1 value for adolescents aged 13–17 at the two centers was below the national median value. The two CFCs participated in the pilot phase of the QIP PHARE-M [6] launched in France in 2011. Their common objective was the improvement of the median FEV1 value of their adolescent patients. The 2 teams decided to work on the psychosocial factors that could affect the respiratory status of these patients and on strengthening these patients’ psychosocial skills in connection with the actions already undertaken as part of their therapeutic education programs. This article seeks to assess in 2015 the effects of the actions implemented in the two CFCs during the QIP and particularly their impact on the FEV1 value in their adolescent cohorts.

## Methods

The methodology of the PHARE-M QIP consisted of:

- the constitution of a quality team in the CFC: “lead” physician, nurse, psychologist, physiotherapist, dietician, and a patient’s parent;
- the participation of the quality teams in 4 training meetings organized by the PHARE-M national team;
- the analysis of the CF center functioning according to the 5Ps assessment: Purpose, Patients, Professionals, Processes, and Patterns;
- the adoption of an improvement theme expressed by a goal on a patient outcome for a population of at-risk patients and a deadline to achieve it;
- the identification of leverage factors and barriers to attain this goal written on a fishbone or cause and effect diagram;
- the definition of PDSA cycles to implement change actions and measure their results on secondary indicators.

### Experience at the Nantes CFC

#### Local context and method

**Fig. 1:**
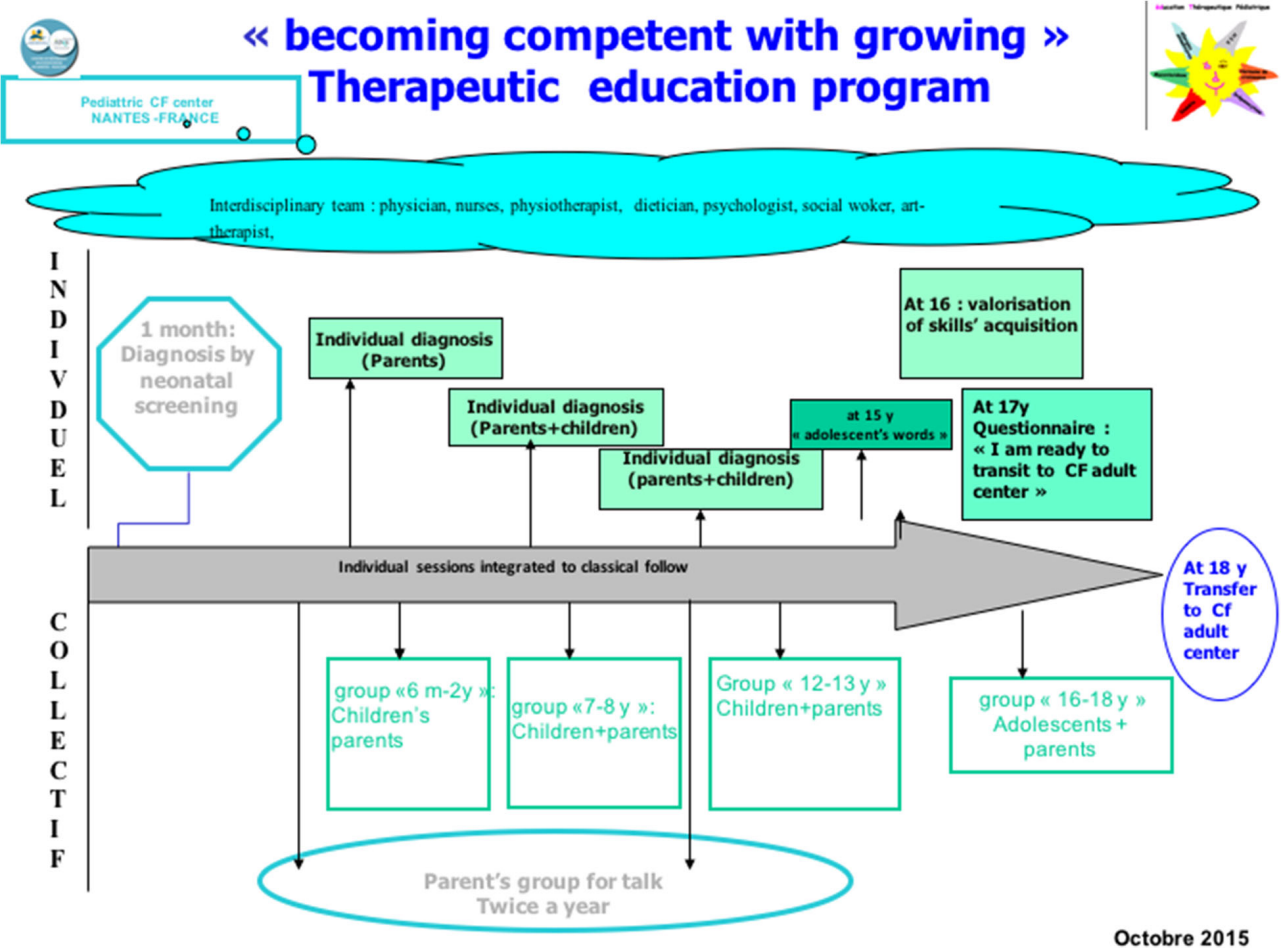
“Becoming competent when growing up with cystic fibrosis” program

Located on the west coast of France, our CFC follows around 1 hundred children aged 1 month to 18 years. Most professionals in our multidisciplinary team have been working at the CFC for several years. Furthermore, the head physician is responsible for promoting and developing a national therapeutic education program in cystic fibrosis. In 2006, our CFC established a well-structured therapeutic education program entitled “Becoming competent when growing up with cystic fibrosis” [6]. This program consists of individual therapeutic education sessions, incorporated into the children’s periodic clinic visits, and of group sessions. The objectives of the sessions are chosen based on parents’ and children’s skills assessment so that they may be centered on the needs identified. The skills to be acquired include self-care and psychosocial skills (Fig. 1).

**Fig. 2:**
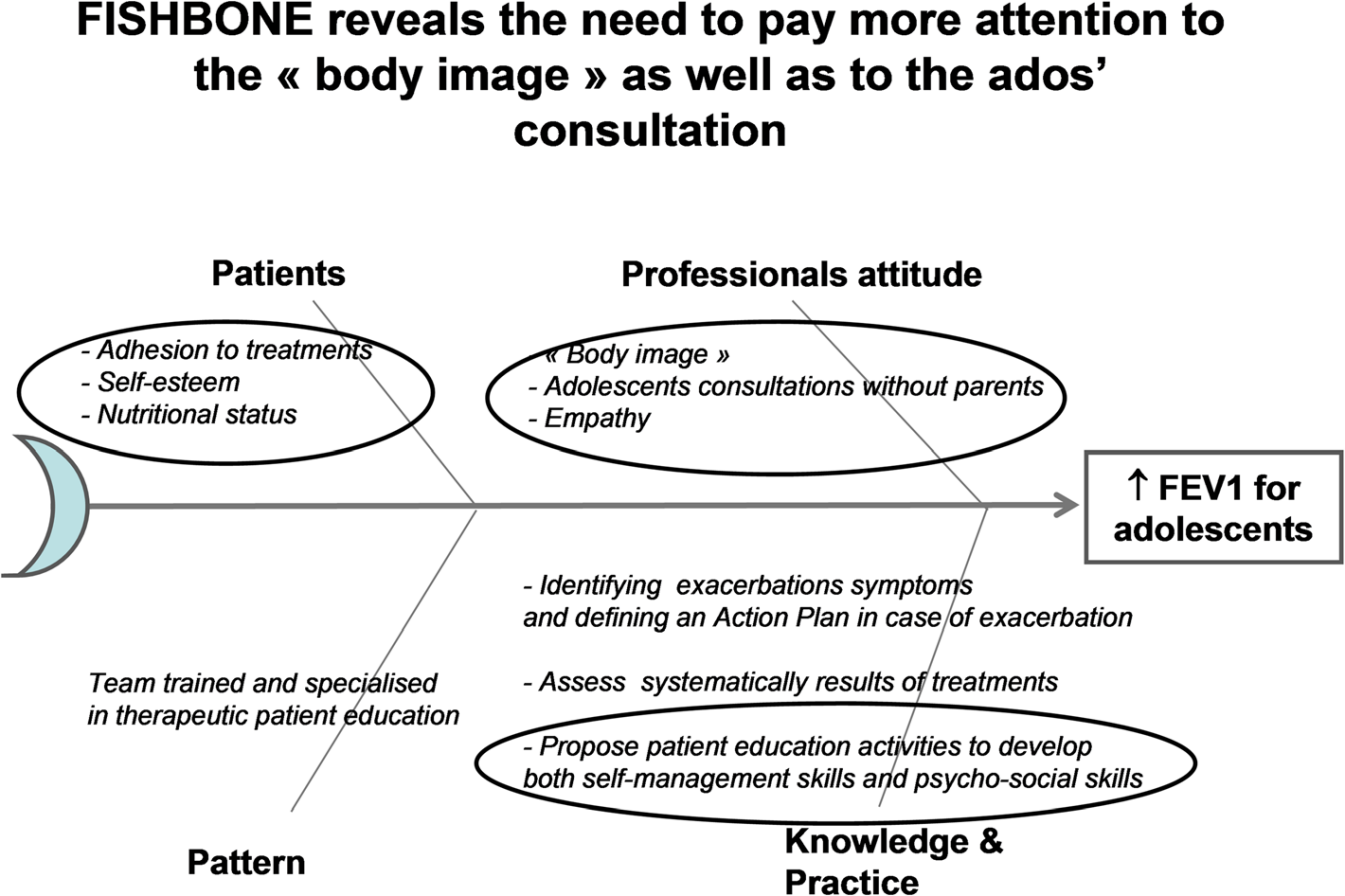
Nantes CFC fishbone diagram

A parent of an adolescent and a quality engineer from the quality department of the Nantes University Hospital were included in our PHARE-M quality team. At the first PHARE-M training session, the quality team set up the following goal: “to improve the median FEV1 value from 78% in 2011 to 85% in 2015 for adolescents aged 15-18”. In addition to the patient outcomes analysis, we conducted 2 satisfaction surveys: one among the CFC’s parents and one among the professionals. Our fishbone listed the causes and levers regarding our improvement goal (Fig. 2). The team decided to prioritize the focus on self-esteem, body image, relationship between the healthcare providers and the adolescent, their motivation for self-management, early detection of pulmonary exacerbations and their access to leisure and sports activities. We listed avenues for improvement in each of these areas and assigned them to every professional in the multidisciplinary team:

- For the physiotherapist, focus on the patient’s attentiveness to their bodily sensations, involving them in their drainage and postural development.
- For the coordinating nurse, focus on the adolescent rather than their parents during the clinic visit, programming the next visit with them and supporting them in achieving their own projects at school, on vacations or in the community.
- For the psychologist, assessing and reinforcing their self-esteem, helping them manage their relationships in their community and the changes inherent to adolescence. The adolescents were also asked to respond to an anxiety/depression/coping questionnaire and the CFQ-R questionnaire.
- For the dietician, assessment of energy expenditure, nutrition regimens and their digestive symptoms.
- For the social worker, socio-economic and cultural assessment in order to facilitate their access to sports and leisure.
- For the art therapist, in connection with the psychologist, improving their self-esteem through creative activities.
- For the physician, a systematic discussion with the adolescent alone, working on the management of their exacerbations, checking their vaccinations, identifying possible issues with tobacco and alcohol and talking about fertility and sexuality.

We determined indicators to be followed up throughout the year in an Excel workbook in the patient record. At the weekly multidisciplinary staff meeting, the professionals scheduled the clinic visit program for the adolescents coming next week according to the needs identified. Every month, the quality team meeting allowed to discuss the indicators and their traceability in the electronic patient record. Annually the quality team analyzed their relevance and the adjustments to be made.

## Results

### Impact on the patients

**Fig. 3:**
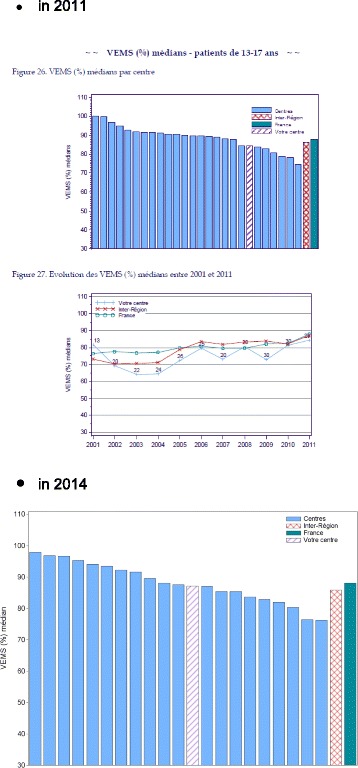
Evolution of Nantes FEV1% ranking for patients aged 13–17

The median FEV1 of adolescents aged 15–18 followed up at Nantes CFC and enrolled in the PHARE-M (*n* = 26) went from 78% in 2011 to 90% in 2015 (Table 1). In addition to the above values, our center also improved its national ranking among all French CFCs, as showed in the histograms issued by the Patient Registry, moving from the bottom third to a central position (Fig. 3).

**Table 1:** Evolution of the median and mean FEV1% of the cohort of adolescents aged 15–18 at the pediatric CFC of Nantes

	2011 N = 26	2012 N = 23	2013 *N* = 23	2014 N = 23	2015 *N* = 26
Mean FEV1	78%	85%	85%	82%	86%
Median FEV1	75%	86%	87%	82%	90%

The adolescents’ responses to the questionnaire on quality of life/coping/anxiety/depression showed that quality of life was good for most of them (average score of 150). Half of the patients did not have anxiety/depression (score below 7), 40% were considered to be “uncertain” (score between 8 and 10), and two were “certain” (score above 11) [7].

The adolescents’ satisfaction was demonstrated through an interview with professionals, or through their involvement in the illustration of the CFC bulletin with the art therapist’s support.

#### Impact on the team and the process of care

**Fig. 4:**
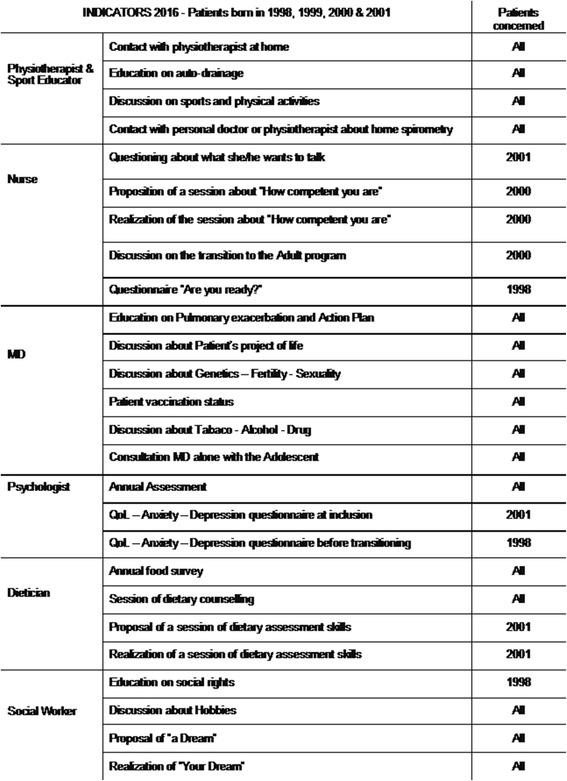
Nantes CFC 2016 indicators

This structured QI project was well received by the team already used to “working together” in therapeutic education group sessions. Professionals expressed their satisfaction in working together on new actions charged with dynamism. Each professional being responsible for a given set of indicators, this led to refine each one’s role and refrain from overlapping during the clinic visit, asking the patient/parent the same questions multiple times. Over the 3 years, the secondary indicators were gradually adjusted and became more precise and more numerous (Fig. 4). The process of care became standardized and was regularly assessed. Progressively, the new process of care was generalized to all the patients followed at the center. Some issues were raised related to the mesosystem level, such as a lack of consultation rooms and slots, and work is going on with the administration to solve these difficulties.

#### Participation of a parent of an adolescent

At first a mother of a 13-year-old child was involved in the program. She participated in our meetings and gave her point of view on the indicators. She stressed that she was speaking in her own name and not on behalf of the parents’ group. After a year, she wished to stop her participation and a mother of an 11-year-old adolescent replaced her, who happened to be a quality engineer. Her contribution from both the perspective of a mother and a professional is still going on.

### Experience at the Paris Robert Debré CFC

#### Local context and method

The Paris (Robert Debré) CFC is a pediatric CFC in Île-de-France following around 170 patients. Since newborn screening was established, the Robert Debré CFC has been managing patients diagnosed with CF from the northeast area of Paris. The families are socially and culturally diverse, mirroring the territory in which the CFC operates. They come from 25 different countries, and many of them are in a difficult or even precarious socioeconomic situation.

Therapeutic patient education (TPE) has been developed at the CFC since 2005. The TPE program has been gradually formalized and strengthened, and received an authorization from the Health Regional Agency in 2011. The TPE program develops along the childhood and the adolescence ages, with skills assessment phases alternating with educational sessions. The sessions offered are most often individual sessions, but group sessions are also organized at certain ages: parents of young children (aged 1–3); children themselves near the end of elementary school and their parents.

**Fig. 5:**
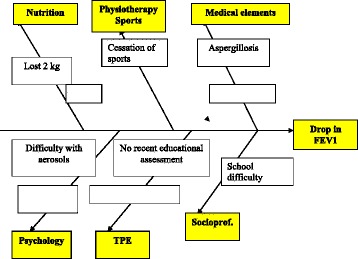
Cause-and-effect diagram for a patient at the Robert Debré CFC

The 5P analysis showed our center’s patterns: a stable and motivated team; a TPE program that operates smoothly; a multicultural population; and patients with a good nutritional status. Our position in the bottom quarter of the histograms for the FEV1% of patients aged 13–17 in the 2011 patient registry report led us to set the goal of improving the FEV1% value for these adolescents by 5% by 2013 and reaching a median FEV1 value ≥85% by 2015. In order to achieve this goal, the quality team chose to develop patient-centered actions and to target firstly the adolescents aged 13–17 with an FEV1 below 80%. Thus we used the fishbone tool to analyze, not a system or a process, but the situation of each patient individually in order to identify the personal factors that could negatively influence their respiratory status. These adolescents had individual interviews with different professionals at the CFC. Experiences were then reflected upon at a multidisciplinary staff meeting bringing together physicians, coordinating nurses, physiotherapists, a dietician, a psychologist, and a social worker. Discussions among professionals enabled to identify the factors that could impact the patient’s FEV1 value and to build a fishbone diagram displaying the barriers or difficulties in different areas: medical factors, nutrition, physiotherapy and sports, psychological or social factors, TPE… as illustrated on a patient example in Fig. 5.

**Fig. 6:**
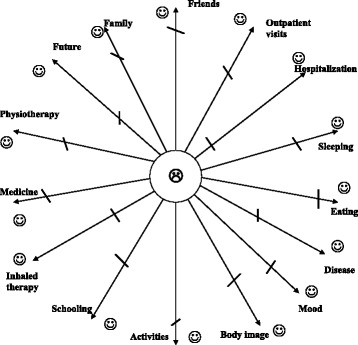
“Feeling star”, psychosocial self-assessment tool from the Robert Debré CFC

Social and psychological factors are shown of particularly importance in adolescence [8]. The psychologist decided to structure the interviews with adolescents and explore the different areas of their lives more comprehensively. As few tools are available, except the quality-of-life questionnaires, she created an educational assessment tool (Fig. 6) centered on adolescent’s “feelings” in connection with the various aspects of their life such as family, physiotherapy, sleep, meals, hospital, body image, friends, medications, future. This tool is as a star with a dozen branches, each of them representing one aspect. During a “face-to-face” interview, the adolescent placed an X on each branch corresponding to their level of “well-being” or “dissatisfaction” or “sadness” for each item. Based on this visual appraisal, the interview continued with open-ended questions to clarify the reasons for satisfaction or discontent. The social worker also met with each adolescent individually for a review of their situation and social needs, concerning their family and its resources, cultural background, identification of carers, sports activities, possibility of going on holidays, existence of a personal project or a dream.

The multidisciplinary team imagined for each adolescent personalized strategies based on the difficulties identified. These proposals were discussed with the adolescents, to build with them concrete actions such as relaxation sessions offered to very anxious adolescents, support in schooling, family mediation, help in expressing and fulfilling a dream. Motivational interviews took place to foster adherence to treatments and to help the adolescents develop their own solutions to overcome the difficulties encountered [9]. The main objective of improving the FEV1 of adolescents aged 13–17 was thus supplemented by other indicators related to the secondary objectives:

- to develop sports activities with the intervention of an APA teacher, quantify the patient’s physical activity, encourage the patient, and assess him/her regularly;
- to increase bronchial drainage thanks to instrumental aids;
- to strengthen self-management and psychosocial skills through their participation in the therapeutic patient education (TPE) program;
- to help the patient express and fulfil a dream or a project.

The FEV1% value and the secondary objectives were the indicators followed for the patients during periodic clinic visits.

## Results

### Impact on the patients

When the PHARE-M program started, 40 adolescents aged 13–17 were followed up at the CFC. Among them, 18 had an FEV1 value below 80%.

For each of them, we made an analysis in a multidisciplinary staff meeting, prepared a cause-and-effect diagram and implemented a personalized action plan. All these patients met with the psychologist for an interview with the “feelings star” tool. This psychosocial self-assessment tool (Fig. 6) highlighted certain problems and needs, especially in less-often explored areas such as sleep and body image. It also allowed care adherence difficulties to be addressed. The example of a patient’s self-assessment with the feelings star is showed in Fig. 6.

**Fig. 7:**
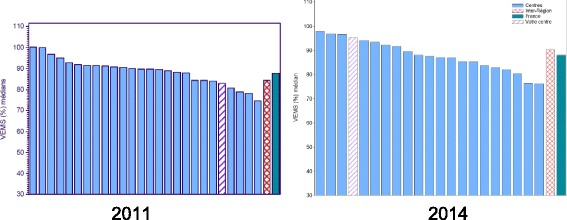
Ranking of Paris R. Debré CFC for adolescents aged 13–17 in 2011 and 2014

At the end of 2015, the main objective of improving pulmonary function was achieved and even exceeded, with a progressive increase in the FEV1% value of the open cohort of patients aged 13–17 (Table 2). However, it is necessary to notice the variability of patients’ evolutions, since some patients improved while others deteriorated during the study. Concomitantly, the rank of our CFC in the national registry improved, moving from the bottom quarter in 2011 to the top quarter in 2015 (Fig. 7).

**Table 2:** Evolution of the FEV1% of the cohort aged 13–17 (R. Debré pediatric CFC)

	2011 *N* = 40	2012 *N* = 35	2013 *N* = 36	2014 *N* = 39	2015 *N* = 38
Mean FEV1	81.7%	84.1%	91.7%	92%	90.5%
Median FEV1	84%	89%	95%	97%	92%

For the secondary objectives, the results varied: by the end of 2013, the 18 adolescents with an FEV1 below 80% had benefited from TPE sessions; 14 out of 18 had been trained in the use of instrumental aids for respiratory physiotherapy and 15 out of 18 had made an assessment of their sports activity and coaching with an APA teacher. But only 5 out of 18 patients achieved the objective “fulfilling a dream”. The APA teacher’s intervention led to an assessment concerning patient satisfaction, implementation of advice given, and possible changes in behavior. This study showed a high satisfaction score among patients (8.1/10); a perceived benefit in terms of a decrease in dyspnea and fatigue; better attention to hydration; and an increase in sports club registrations, which went from 56 to 65%. The use of instrumental aids and the benefit perceived by the patient are still being assessed. Finally, these actions, first initiated for adolescents aged 13–17 with an FEV1 value below 80%, were then extended to this entire age range, regardless of one’s FEV1 value.

### Impact on the team and the process of care

The PHARE-M was positively received by the team and built a positive team dynamic. The team’s cohesion was strengthened. This program empowered each professional and recognized their specific skills. Thanks to the program, the entire team better recognized the psychosocial impact of the disease on the adolescents. The psychologist’s and social worker’s roles within the team were particularly highlighted, with their increased involvement and participation in the multidisciplinary staff meetings. Finally, the relationship between the team and the patient was often improved, with the team gaining a more comprehensive vision of the patient and their needs, especially in the psychosocial area.

A more structured multidisciplinary analysis of the patient’s situation was implemented. The patient-centered approach, already developed within the framework of the TPE program, was generalized. New tools were developed, such as the “feelings star”. As the initial analysis showed that the adolescents were unaware of the social worker’s role as a “resource person”, a specific session was established for all patients with her, to discuss patient rights at school or at the university and the possible status of disabled worker.

## Discussion

The two CFCs in Paris (Robert Debré) and Nantes chose to improve the FEV1 in adolescents by strengthening their psychosocial skills through a patient-centered approach, in connection with their respective therapeutic education programs. The goals were achieved for both teams in terms of patient outcomes and satisfaction, and in terms of teams’ functioning, interdisciplinary work and development of innovative actions. In the two centers, the FEV1% stabilized at a median value of 90% for the population of adolescents after 4 years. The improvement in the national ranking of both CFCs also suggests an acceleration of their progression compared to the overall national improvement in FEV1% for this age range.

### Limitations

It is difficult to attribute these results exclusively to the PHARE-M, as an overall improvement was observed in the respiratory function of CF patients (cf. the annual French national registry data [10], (http://vaincrelamuco.org/sites/default/files/rapport_registre_2014.pdf). Furthermore, our cohorts were open, not very numerous and heterogeneous, and various individual evolutions were observed.

### Other benefits induced by PHARE-M

Within the framework of the PHARE-M QIP, the participation of a quality engineer from the quality department in Nantes Hospital and the involvement of a parent were key assets for the team. The PHARE-M methodology focuses on the follow-up of indicators, in “real time” and not exclusively based on registry data issued with a lag of one or more year. Assessing the suitability of the indicators lead to readjusting them on a regular basis. Thanks to that, the quality improvement program becomes a process that continues over time [11]. Certain difficulties were noted by the teams regarding information traceability in the patient record, the regularity of the “quality” staff meetings, and the teams’ long-term motivation. The annual Quality Improvement experience-sharing days, organized after the training year for the CFCs having participated in PHARE-M, seem essential to maintain a dynamic of continuous quality improvement.

### Impact on the quality of care

Over the years, improvements in CF care have been made at our CFCs. The role of the social worker and the psychologist became more important for the adolescents. New professionals were incorporated into our teams, with their specific skills: a physical activity teacher at Robert Debré and an art therapist at Nantes. When incorporated into patient care with specific follow-up indicators, creative activities can improve adherence to the treatment thanks to the increase in adolescents’ satisfaction with the team [12]. Among the adolescents followed up at the Nantes CFC, quality of life was most often good and anxiety scores were most often normal; this differed from the results found in the literature [13, 14].

### Synergy between QIP and patient education

The physicians in charge of the PHARE-M at these two centers were also in charge of TPE programs and members of the GETHEM French national working group for the development of TPE in cystic fibrosis in France. Work and reflection on the adolescents’ FEV1 led to identify the skills to be strengthened within this population. Their self-management of care knowledge and skills seemed generally satisfactory; however, their psychosocial skills were often fragile and deserved to be strengthened before the transition to the adult program. At Nantes, this transition is structured at key times, such as discussions about transition since 15 years of age and the “Are you ready?” assessment inspired by the Canadian questionnaire (http://www.bcchildrens.ca/Cystic-Fibrosis-site/Documents/ReadinesstoTransitionquestionnaire2008.pdf), 6 months to 1 year before the transitioning process. At Robert Debré, “pre-transition educational assessment” had been used for several years between 16 and 18 years of age leading to educational sessions according to the needs identified. The two teams created a common adolescent assessment approach, based on the existing adult model [15]. This key period for patient follow-up was the subject of a specific quality improvement program in California, United States, in which both teams found similar approaches to determine whether an adolescent is ready to transition [16]. Moreover, some psychological needs were identified in the parents regarding empowering their adolescents, supporting them, managing emotions… It is thus essential to involve them in the transition process. Helping the parents support the adolescent, redefine their role and express their fears and hopes are important objectives in the transition process and may help them for a quiet transition to adulthood with CF [17].

## Conclusion

The PHARE-M provides tools and a methodology which structures the QIP towards the optimization of the process of care. The experience of the two CFCs shows that PHARE-M relies on the teams’ culture, in this case, the educational programs for the development of the adolescents’ skills. Through the PHARE-M, the CFCs combined a systematic approach on processes and an individualized approach centered on each adolescent. This “patient-centered quality process” maximized the QIP’s effects and allowed consideration of the patient’s needs. The strong involvement of psychologists and social workers in the TPE programs and the PHARE-M QIP strongly benefited to the adolescents and their families.
